# Data on heavy metal levels (Cd, Co, and Cu) in wheat grains cultured in Dashtestan County, Iran

**DOI:** 10.1016/j.dib.2017.08.012

**Published:** 2017-08-09

**Authors:** Abdolhamid Esmaili, Vahid Noroozi Karbasdehi, Reza Saeedi, Mohammad Javad Mohammadi, Tayebeh Sobhani, Sina Dobaradaran

**Affiliations:** a Department of Pathology, School of Medicine, Bushehr University of Medical Sciences, Bushehr, Iran; b Department of Environmental Health Engineering, Faculty of Health, Bushehr University of Medical Sciences, Bushehr, Iran; c Department of Health Sciences, School of Health, Safety and Environment, Shahid Beheshti University of Medical Sciences, Tehran, Iran; d Abadan school of Medical Sciences, Abadan, Iran; e The Persian Gulf Marine Biotechnology Research Center, The Persian Gulf Biomedical Sciences Research Institute, Bushehr University of Medical Sciences, Bushehr, Iran; f Systems Environmental Health, Oil, Gas and Energy Research Center, The Persian Gulf Biomedical Sciences Research Institute, Bushehr University of Medical Sciences, Bushehr, Iran

**Keywords:** Heavy metal, Wheat grains, Dashtestan county, Food sanitary

## Abstract

Due to importance of wheat as the most popular food, in this data article, we determined the accumulation of heavy metal levels including Cd, Co, and Cu in wheat grains in Dashtestan county, Iran. The concentration levels of heavy metals in wheat grains cultured were determined by Flame Atomic Absorption Spectrometry (FAAS).^1^

**Specifications Table**

**Table:** 

*Subject area*	*Chemistry*
*More specific subject area*	*Food sanitary*
*Type of data*	*Table*
*How data was acquired*	*Flame Atomic Absorption Spectrometry (Varian AA240 model, Australia)*
*Data format*	*Raw, analyzed*
*Experimental factors*	*Wheat grain samples were washed with tap water to remove any attached particles, rinsed three times with distilled water, and then dried at 38 °C till constant weight. Dried samples were ground by using a stainless steel grinder (<0.25 mm) for heavy metal analysis. A portion of the dry wheat grains powder were digested in a mixture of HNO*_*3*_*–HClO*_*4*_*–H*_*2*_*SO*_*4*_*acids.*
*Experimental features*	*Evaluate the metal contents of Cd, Co, and Cu in wheat grains in Dashtestan county, Iran*
*Data source location*	*Bushehr, Dashtestan county, Iran*
*Data accessibility*	*Data is with this article.*

**Value of the data**

• Data can be used as a base-line data for metal concentration levels in wheat grains.
• Data shown here can be useful for policy makers, managers, and all related stakeholders, companies, agencies, and institutes working in the fields of food sanitary by imposing proper measures to protect soil from pollutants.
• Data shown here may serve as benchmarks for other groups working or studying in the field of toxicology, soils amended with domestic sewage or irrigated with industrial effluents.

## 1 Data

The data in Table 1 show that Cd, and Co level were below limit of detection (BLD) in all wheat samples, but the mean concentration levels of Cu was 0.501 with a range of 0.223–0.849 µg/g, and the content level of moisture in wheat samples ranged from 10.15–14.88 (Mean: 11.51%). The measured detection limit values for Cd, Co and Cu were 0.0047, 0.015 and 0.0055 µg/g respectively. Each sample were measured three times and average were reported.

**Table 1:** The content levels of heavy metals (µg/g) and moisture (%) in wheat grain samples.

***Region***	***Number of samples***	***Samples***	***Moisture (%)***	***Cd (µg/g)***	***Co (µg/g)***	***Cu (µg/g)***
*Tang Eram*	*4*	*1*	*10.15*	*BLD*^a^	*BLD*	*0.65*
		*2*	*11.31*	*BLD*	*BLD*	*0.509*
		*3*	*11.25*	*BLD*	*BLD*	*0.223*
		*4*	*14.88*	*BLD*	*BLD*	*0.405*
*Sadabad*	*4*	*1*	*11.81*	*BLD*	*BLD*	*0.633*
		*2*	*11.15*	*BLD*	*BLD*	*0.540*
		*3*	*11.07*	*BLD*	*BLD*	*0.499*
		*4*	*10.16*	*BLD*	*BLD*	*0.592*
*Shaban Kareh*	*4*	*1*	*11.57*	*BLD*	*BLD*	*0.849*
		*2*	*11.97*	*BLD*	*BLD*	*0.435*
		*3*	*11.29*	*BLD*	*BLD*	*0.435*
		*4*	*11.45*	*BLD*	*BLD*	*0.245*
*Mean*	*12*	–	*11.51*	*BLD*	*BLD*	*0.501*
*Maximum*		–	*14.88*	*BLD*	*BLD*	*0.849*
*minimum*		–	*10.15*	*BLD*	*BLD*	*0.223*
*Detection limit*		–	–	*0.0047*	*0.015*	*0.0055*

a BLD: Below limit of detection

## 2 Experimental design, materials and methods

### 2.1 Study area description

**Fig. 1:**
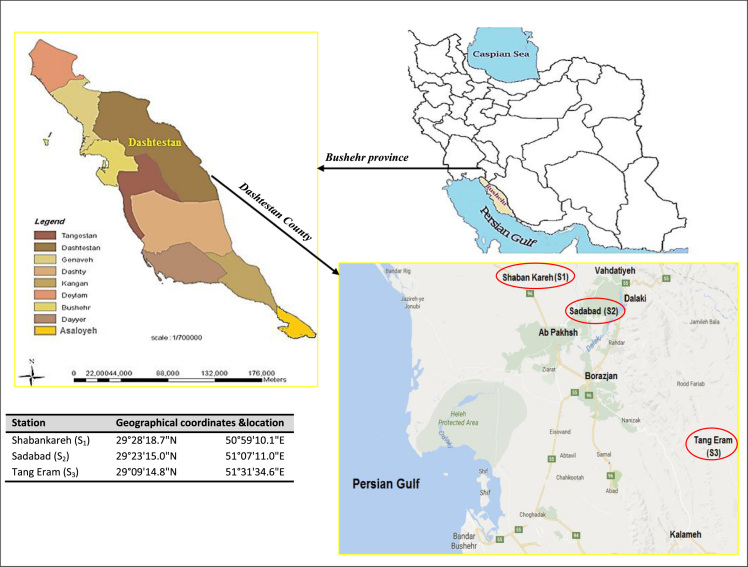
Geographic coordinates and location of sampling station.

Dashtestan County is the biggest county in Bushehr Province, in south west of Iran. This county has the first rank in production of date palm and cereals in Bushehr Province. The capital of the county is Borazjan. In this study, three important regions in wheat production including Shabankareh, Sadabad, and Tang Eram were selected as sampling points (Fig. 1).

### 2.2 Sample collection and preparation

Twelve sample were collected in three agricultural areas in Dashtestan county (each site 4 times) just before wheat harvest. Nitrogen fertilizers were used on these grounds. Wheat plants at grain maturity (just before harvest) were randomly chosen within a 5 m×5 m square, were cut with scissors at a height above 10 cm from the soil surface. The wheat samples were air dried for 8 days, and then put into labeled bags and transported to the lab. In the laboratory, grain samples were washed with tap water for 60 min to remove any attached particles, and rinsed three times with distilled water, and oven dried at 38 °C till constant weight. Dried samples were ground using a stainless steel grinder (<0.25 mm) for heavy metal analysis.

### 2.3 Reagents

All the employed oxidants and mineral acids including HNO_3_, H_2_SO_4_, and HClO_4_ were of suprapure quality (Merck, Darmstadt, Germany).

### 2.4 Digestion and analytical procedures

A 2 g dried samples were crushed in a mortar and ashed in a muffle furnace at 450^◦^C for 6 h [1]. If the ashes were not completely white, 2 mL of concentrated HNO_3_ were added and the mixture was heated to boiling point on an electric plate heater until the formation of nitrous fumes had stopped [2]. Then, the ashes were returned to the muffle at 450 °C for a further 2 h. Finally, the white ashes were digested in a mixture of HNO_3_–HClO_4_–H_2_SO_4_ acids (10 ml 70% HClO_4_, 32 ml 10% HNO_3_, and 5 ml 90% H_2_SO_4_) according to standard analytical procedures [3], [4]. A Flame Atomic Absorption Spectrometry (FAAS, Varian AA240, Australia) [5], [6], [7], [8] was used to determine the content levels of Cd, Co, and Cu.
